# What Is the Profile of Overweight Individuals Who Are Unsuccessful Responders to a Low-Energy Diet? A PREVIEW Sub-study

**DOI:** 10.3389/fnut.2021.707682

**Published:** 2021-11-02

**Authors:** Angelo Tremblay, Mikael Fogelholm, Elli Jalo, Margriet S. Westerterp-Plantenga, Tanja C. Adam, Maija Huttunen-Lenz, Gareth Stratton, Tony Lam, Teodora Handjieva-Darlenska, Svetoslav Handjiev, J. Alfredo Martinez, Ian A. Macdonald, Elizabeth J. Simpson, Jennie Brand-Miller, Roslyn Muirhead, Sally D. Poppitt, Marta P. Silvestre, Thomas M. Larsen, Pia Siig Vestentoft, Wolfgang Schlicht, Vicky Drapeau, Anne Raben

**Affiliations:** ^1^Department of Kinesiology, Faculty of Medicine, Laval University, Quebec, QC, Canada; ^2^Department of Food and Nutrition, University of Helsinki, Helsinki, Finland; ^3^Department of Nutrition and Movement Sciences, Maastricht University, Maastricht, Netherlands; ^4^Institute of Nursing Science, University of Education, Schwäbisch Gmünd, Germany; ^5^School of Sport and Exercise Sciences A-STEM Research Centre, Swansea University, Swansea, United Kingdom; ^6^NetUnion sarl, Lausanne, Switzerland; ^7^Department of Pharmacology and Toxicology, Medical University of Sofia, Sofia, Bulgaria; ^8^Department of Nutrition Research, University of Navarra, Pamplona, Spain; ^9^CIBERobn, Instituto de Salude Carlos III, Madrid Spain and IMDEA Madrid, Madrid, Spain; ^10^School of Life Sciences, Faculty of Medicine and Health Sciences, University of Nottingham, Nottingham, United Kingdom; ^11^Charles Perkins Centre and School of Life and Environmental Sciences, University of Sydney, Sydney, NSW, Australia; ^12^Human Nutrition Unit, School of Biological Sciences, University of Auckland, Auckland, New Zealand; ^13^Centro de Investigaçao em Tecnologias e Serciços de Saûde (CINTESIS), NOVA Medical School NOVA University of Lisbon, Lisbon, Portugal; ^14^Department of Nutrition, Exercise and Sports, Faculty of Science, University of Copenhagen, Copenhagen, Denmark; ^15^Exercise and Health Sciences, University of Stuggart, Stuggart, Germany; ^16^Department of Physical Education, Faculty of Educational Sciences, Laval University, Quebec, QC, Canada; ^17^Department of Nutrition, Exercise and Sports, Faculty of Science, University of Copenhagen, Copenhagen and Steno Diabetes Center, Gentofte, Denmark

**Keywords:** obesity, behavior, energy, hunger, appetite, sleep

## Abstract

This study was performed to evaluate the profile of overweight individuals with pre-diabetes enrolled in PREVIEW who were unable to achieve a body weight loss of ≥8% of the baseline value in response to a 2-month low-energy diet (LED). Their baseline profile reflected potential stress-related vulnerability that predicted a reduced response of body weight to a LED programme. The mean daily energy deficit maintained by unsuccessful weight responders of both sexes was less than the estimated level in successful female (656 vs. 1,299 kcal, *p* < 0.01) and male (815 vs. 1,659 kcal, *p* < 0.01) responders. Despite this smaller energy deficit, unsuccessful responders displayed less favorable changes in susceptibility to hunger and appetite sensations. They also did not benefit from the intervention regarding the ability to improve sleep quality. In summary, these results show that some individuals display a behavioral vulnerability which may reduce the ability to lose weight in response to a diet-based weight loss program. They also suggest that this vulnerability may be accentuated by a prolonged diet restriction.

## Introduction

The clinical and research experience in obesity management shows that there are substantial variations in the response to diet and/or exercise-based interventions aiming at the improvement of body composition and metabolic fitness in individuals with obesity (1). In some cases, these variations may reveal a clear deterioration of body homeostasis. In this context which supposedly has the potential to improve the condition of everybody, these negative changes have been traditionally attributed to a lack of compliance although experimental data show that the failure to succeed may be attributable to other factors. In the HERITAGE Project, Bouchard et al. (2) examined the profile of adverse responders to exercise-training under highly standardized intervention conditions. Non-compliance was not possible in this study since the ergocycle exercise prescription was individually pre-programmed and closely supervised by an exercise specialist. Despite this careful monitoring, the investigators detected responders for whom an adverse change greater than the technical error was found in systolic blood pressure as well as fasting plasma insulin, HDL-cholesterol, and triglycerides. Most importantly, about 7% of participants experienced a deterioration for two or more risk factors.

In a more recent study, we divided a small cohort of women with obesity in thirds based on their body weight response to a diet-based weight-reducing intervention (3). In the upper third, the reduction in body weight was conform to the expected diet-induced decrease in energy intake whereas no mean change in body weight was observed in the lowest third which included 11 body weight gainers. Two findings emerged from this study: (1) The variation in body weight response could not have been predicted from the baseline profile of participants which was based on nutritional, physiological, and behavioral measurements; (2) The lowest third, i.e., the non-responders to diet, displayed less favorable changes for energy intake, susceptibility to hunger as well as sleep duration and quality compared to the highest third. In addition, resting metabolic rate decreased more than the predicted change in the non-responders whereas the opposite trend was observed in the good responders.

These observations are concordant with those reported in the clinical trial of Sacks et al. (1) who tested the impact of reduced-energy diets differing by their macronutrient composition on body weight loss in individuals with overweight. The results showed that mean weight loss was comparable among the four tested diets and that attendance at group sessions was strongly associated with weight loss. Interestingly, the illustration of individual changes in body weight for each diet showed that there were weight gainers even among participants attending a high number of sessions.

The completion of the PREVIEW Study (4) offers an opportunity to pursue the characterization of the profile of individuals who seem unable to achieve an expected weight loss in response to a controlled low-energy-diet (LED)-based weight-reducing program. The 3-year intervention was initiated by a 2-month phase of rapid weight loss induced by the use of LED (5, 6). To be eligible for the subsequent weight-maintenance intervention of 34 months, each participant had to achieve a weight loss of at least 8% of baseline body weight. However, there were individuals who were excluded from the weight-maintenance phase due to insufficient weight loss. The profile of these apparently low responders is the main focus of the present study that is aimed to determine if the baseline level and/or the response of the morphological, metabolic, and behavioral profile of PREVIEW participants can differentiate the successful responders having lost at least 8% of their baseline body weight during Phase 1 from the unsuccessful responders who were unable to achieve this weight loss. In broader terms, in this paper we aim to study determinants of poor response to LED in individuals who are overweight or obese and with prediabetes.

## Methods

### Design and Intervention

PREVIEW was a research collaborative project that was aimed to evaluate the effects of diet and physical activity programmes as well as their interactions on body weight and health-related outcomes. It included a large multicentre clinical trial (clinicaltrials.gov NCT01777893) that was conducted at eight intervention sites: University of Copenhagen (Denmark), University of Helsinki (Finland), University of Maastricht (the Netherlands), University of Nottingham (UK), University of Navarra (Spain), Medical University of Sofia (Bulgaria), University of Sydney (Australia) and University of Auckland (New Zealand). The 36-month intervention tested in this project was initiated by a 2-month phase of rapid weight loss achieved through use of a commercial LED.

Beyond its contribution to the global PREVIEW intervention, the first phase of the project was also used as a qualification period regarding the ability to pursue the second phase of the protocol (34-month weight maintenance). The participants who lost a minimum of 8% of their baseline body weight were accepted in the second phase whereas those who attended end of LED assessment but who did not achieve this threshold were excluded from the study. In this paper, these participants are classified as successful (S) and unsuccessful (U) responders, respectively.

The Cambridge Weight Plan (Northants, UK) was used to promote a negative energy balance and weight loss during the 8 weeks of this phase of the project. This diet was a LED which had an energy content of 3,400 kJ (800 kcal) with a macronutrient composition of 15–20, 35–40, and 45–50% energy as fat, protein, and carbohydrate, respectively. The daily diet plan comprised 4 × 40 g meal replacement sachets (The Cambridge Weight Plan, Northants, UK); three were dissolved in low-fat milk or similar lactose-free alternatives and one was dissolved in 250 mL water. Energy-free drinks were permitted as well as a maximum of 400 g non-starchy, low-carbohydrate vegetables, e.g., lettuce, celery, broccoli, or cucumber.

The compliance of participants was facilitated by the attendance at group visits at weeks 2, 4, 6, and 8. The sum of these visits and the baseline session was calculated and used as an index of compliance to the intervention. These meetings also allowed a researcher to monitor body weight, record medications, distribute LED sachets, and give dietary and behavioral instructions. No guideline was given about physical activity participation during this weight reduction phase.

The participants who did not complete the 2-month LED intervention were not considered in this study. Therefore, the variables analyzed in this paper were measured before and after the 2-month intervention in all completers of this phase.

### Participants

Male and female adult participants were recruited in the eight intervention centers. As previously described (5), the basic inclusion criteria were: age 25–70 years; BMI>25 kg/m^2^; pre-diabetes confirmed by an OGTT according to the criteria of the American Diabetes Association (7). The complete list of inclusion and exclusion criteria as well as the procedures of blood collection have been reported elsewhere (5).

Each participant gave his/her written consent to participate in the study protocol that was approved by the local ethics committee of the eight intervention sites. The PREVIEW procedures were also conform to the relevant rules and guidelines of the Declaration of Helsinki (59th WMA General Assembly, Seoul, Korea, October 2008) and the International Conference on Harmonization for Good Clinical Practice.

### Anthropometric Data and Body Composition

Body weight was measured in fasting state with an empty bladder and wearing underwear or other light clothing. Two measurements were taken to the nearest 0.1 kg. A second series of measurements was performed and the mean value of the two weights was used for analysis. Height was measured without wearing shoes while heels, buttocks and upper part of the back were in contact with a wall-mounted stadiometer. The average of two measurements to the nearest 0.5 cm was used for analysis.

Waist circumference was also assessed in fasting state with an empty bladder. The measurement was made with a tape to the nearest 0.5 cm and was taken at mid-distance between the lower rib and iliac crest. The mean of two measurements was also used for analysis.

The measurement of body composition was preferably performed by dual X-ray absorptiometry, underwater weighing, Bod Pod or deuterium dilution, depending on the availability in each intervention site. When this type of equipment was not available, bioelectrical impedance was then used. In each case, determinations were done according to the manufacturer's instructions under fasting conditions.

### Blood Pressure and Heart Rate

Systolic and diastolic blood pressure was measured with an autonomic device following a 5–10 min rest position. Three measurements to the nearest 1 mm Hg were taken with a 1 min interval. The mean value of the three readings was recorded in the databank. Resting heart rate was also measured in the same session that was held following at least 12 h without high-intensity physical activity, smoking or coffee intake.

### Physical Activity Participation

The Baecke questionnaire (8) was used to evaluate habitual physical activity participation. The questionnaire includes 22 frequency questions which document work, sport and leisure activities corresponding to three indexes.

### Appetite Sensations and Eating Behaviors

Visual analog scales (VAS) were used to estimate appetite sensations in a free-living context at home before the visit in the clinic. For that purpose, participants had to rate their perception of hunger, satiety, fullness, desire to eat, desire to eat something sweet, desire to eat something savory, and thirst on a 100 mm scale. Their validity was demonstrated in the context of diet-based weight-reducing programs (9).

The Three-Factor Eating Questionnaire (TFEQ) (10) was used to measure three dimensions of eating behaviors including cognitive restraint, disinhibition, and hunger, with 51 items. Cognitive restraint is the intent to restrict food intake to control body weight. Disinhibition reflects the susceptibility to overeat whereas hunger measures the susceptibility to hunger feelings. Each of these variables was found to be related to the proneness to overweight in the Quebec Family Study (11).

### Behaviors and Social Environment

Quality of life was assessed with the WHOQOL-BREF (12) which is an abbreviated version of the WHOQOL-100. This tool produces scores related to the four following domains: physical health, psychological, social relationships, and environment.

The Work Ability Index (WAI) (13) identifies factors influencing the intention to continue working. This motivation has been shown to be related to perceived health and social support.

Non-specific perceived stress was measured with the Perceived Stress Scale (PSS) (14) which contains 10 items. An increase in the score reflects an increase in perceived stress.

### Sleep

The Pittsburgh Sleep Quality Index (PSQI) (15) was used to study variations in sleep duration and quality. Specifically, we analyzed answers to Question 4 of this questionnaire which refers to sleep duration. We also report the total PSQI score derived from the 21 items of the questionnaire which reflects sleep quality. Our clinical experience shows that both sleep quality and duration derived from the PSQI are related to the fat loss induced by a diet-based weight-reducing program (16).

### Statistical Analyses

The values are shown as mean ± SD. The statistical comparisons were performed on baseline age-adjusted scores using a two-way ANOVA considering effects of sex, group (successful vs. unsuccessful responders) and sex by group interaction. The same procedure was used for the analyses of changes induced by the LED intervention. Pearson correlation analyses were also performed to evaluate the association between baseline level of some variables and percent body weight loss in all completers of Phase 1. The JMP software (version 15.2.0) was used for the analyses.

## Results

Descriptive characteristics of participants are presented in Table 1. In both men and women, baseline body weight was slightly greater in U than in S weight loss responders although the difference did not reach statistical significance. According to the design of the present analysis, the mean percent weight loss in U responders was substantially smaller than the predetermined criterion of 8% baseline body weight. In S responders, the mean body weight decrease was more than twice that of U responders, be it expressed in absolute or relative values.

**Table 1:** Descriptive characteristics of unsuccessful (U) and successful (S) responders to LED.

**Variable**	**Group**	**Women**	**Men**
Age (yr.)^+^	U S	49.8 ± 12.4^*^51.7 ± 11.2	49.7 ± 13.3 53.8 ± 11.1
Height (m)^+^	U S	1.63 ± 0.071.64 ± 0.07	1.78 ± 0.08 1.77 ± 0.07
Body weight (kg)^+^	U S	96.4 ± 22.494.7 ± 18.3	112.5 ± 26.1 109.5 ± 21.1
Body weight loss (kg)	U S	4.5 ± 2.510.3 ± 2.6	5.3 ± 2.7 13.4 ± 3.5
Percent weight loss	U S	4.7 ± 2.410.9 ± 2.0	4.8 ± 2.3 12.4 ± 2.7

* *Values are means ± SD. + Values obtained at the screening visit*.

*For each variable, the number of subjects was 138 and 1,214 for U and S women, respectively. The corresponding number for men was 53 and 615*.

Among the completers of Phase 1, i.e., the LED intervention, the proportion of U responders was 10.2 and 7.9% for women and men, respectively (Table 1). Table 2 shows that there were several differences in the baseline profile of the two groups of responders. Resting heart rate and self-reported perceived stress were higher in U responders. With respect to eating behaviors, the U responders displayed a higher level of dietary restraint and lower level of disinhibition compared to S responders. It is also relevant to indicate that the fullness level measured by VAS was lower in U than in S responders.

**Table 2:** Baseline profile of the two groups of responders to LED.

**Variable**	**Group**	**Women**	**Men**	**p—sex**	**p—group**	**p—sex × group**
Body weight (kg)	U S	96.2 ± 22.7^*^94.7 ± 18.5	110.9 ± 25.7 109.0 ± 21.3	<0.0001	0.8495	0.8567
Fat-free mass (kg)	U S	51.6 ± 8.7050.4 ± 7.37	71.3 ± 15.0 68.4 ± 9.78	<0.0001	0.0898	0.4417
Fat mass (kg)	U S	44.4 ± 14.143.9 ± 12.6	39.2 ± 14.4 40.2 ± 14.4	<0.0002	0.3776	0.356
Percent fat	U S	45.9 ± 5.7046.4 ± 5.72	35.0 ± 6.60 36.8 ± 6.62	<0.0001	0.0171	0.2093
Waist circumference (cm)	U S	106.5 ± 15.5107.1 ± 13.5	115.5 ± 15.8 116.7 ± 14.2	<0.0001	0.3648	0.7588
Systolic blood pressure (mmHg)	U S	127.4 ± 16.3127.3 ± 16.1	134.2 ± 16.5 133.4 ± 14.7	<0.0001	0.2002	0.502
Diastolic blood pressure (mmHg)	U S	77.6 ± 11.977.0 ± 11.3	82.7 ± 11.8 80.8 ± 9.48	<0.0001	0.1066	0.4045
Heart rate (bpm)	U S	74.0 ± 10.571.3 ± 10.2	73.1 ± 9.70 69.5 ± 11.0	0.1711	0.0031	0.825
TFEQ restraint	U S	9.20 ± 4.378.46 ± 4.06	7.65 ± 4.90 6.87 ± 4.04	<0.0001	0.0067	0.8204
TFEQ disinhibition	U S	9.44 ± 3.419.67 ± 3.50	7.35 ± 2.81 8.20 ± 3.39	<0.0001	0.0184	0.2221
TFEQ hunger	U S	7.06 ± 3.617.20 ± 3.54	6.14 ± 2.90 6.77 ± 3.62	0.0362	0.1148	0.3667
PSS	U S	17.5 ± 6.014.2 ± 6.29	14.5 ± 6.59 12.4 ± 6.03	<0.0001	<0.0001	0.1509
PSQI (n hours)	U S	7.88 ± 1.257.97 ± 1.27	7.80 ± 0.96 7.65 ± 1.19	0.0398	0.4697	0.206
PSQI (total score)	U S	6.97 ± 3.326.61 ± 3.42	6.30 ± 3.36 6.02 ± 3.10	0.0335	0.3655	0.8285
Baecke-work index	U S	2.42 ± 0.742.39 ± 0.77	2.47 ± 0.74 2.38 ± 0.77	0.747	0.6497	0.7758
Baecke-sport index	U S	2.07 ± 0.642.18 ± 0.69	2.41 ± 0.71 2.33 ± 0.73	<0.0001	0.6701	0.0685
Baecke-leisure index	U S	2.44 ± 0.662.60 ± 0.69	2.55 ± 0.64 2.63 ± 0.70	0.3081	0.1204	0.4201
QOL(total score)	U S	25.9 ± 0.6025.8 ± 0.59	25.9 ± 0.40 25.9 ± 0.38	0.4168	0.8111	0.3577
WAI (total score)	U S	38.3 ± 5.1239.6 ± 5.19	41.7 ± 5.30 40.5 ± 4.98	<0.0001	0.7301	0.0232
VAS-hunger (mm)	U S	52.6 ± 26.348.3 ± 22.8	45.9 ± 21.6 48.4 ± 21.2	0.1233	0.8697	0.0455
VAS-satiety (mm)	U S	61.8 ± 24.661.0 ± 20.0	65.1 ± 19.9 61.3 ± 17.8	0.2725	0.1842	0.3862
VAS-fullness (mm)	U S	55.0 ± 29.759.1 ± 22.6	54.1 ± 28.5 61.1 ± 20.1	0.7536	0.0033	0.449
VAS-thirst (mm)	U S	51.4 ± 29.750.3 ± 25.1	52.3 ± 26.3 50.6 ± 20.9	0.7191	0.8146	0.9516
VAS-sweet (mm)	U S	55.5 ± 32.859.2 ± 27.8	49.6 ± 30.5 50.5 ± 26.5	0.0032	0.0769	0.8064
VAS-savory (mm)	U S	55.1 ± 28.659.7 ± 24.5	60.8 ± 26.4 60.9 ± 21.3	0.1029	0.2758	0.2784
VAS-desire to eat (mm)	U S	63.6 ± 25.465.1 ± 20.8	64.9 ± 19.3 61.5 ± 19.2	0.6109	0.9862	0.2472

* *Values are means ± SD. Analyses were performed on scores adjusted for age*.

*Group of responders: U and S refer to unsuccessful and successful, respectively*.

*See Table 1 for sample numbers*.

To ascertain the clinical relevance of differences observed at baseline between the two groups of responders, correlation analyses were performed to evaluate their association with variations in percent weight loss in response to the LED intervention. Table 3 shows that in both women and men, baseline levels of body weight, dietary restraint, perceived stress, and resting heart rate were all negatively associated with percent weight loss, i.e., they predicted a reduced relative decrease in body weight. Finally, this table shows that baseline fullness measured by VAS was positively correlated with the percent change in body weight.

**Table 3:** Correlations between percent body weight loss and baseline level of relevant variables^*^.

**WOMEN**	**Estimate**	**SE**	***P*** **-value**	**R Square**
**Variables**				
Body weight	−0.5214	0.1839	0.0046	0.0059
Restraint	−0.1813	0.041	<0.0001	0.0158
Disinhibition	0.1214	0.0349	<0.0005	0.0096
Perceived stress	−0.3371	0.0621	<0.0001	0.0222
Heart rate	−0.4467	0.0996	<0.0001	0.0147
Fullness	1.2039	0.2291	<0.001	0.0208
**MEN**				
Body weight	−0.8949	0.2469	0.0003	0.0193
Restraint	−0.1738	0.0484	0.0004	0.0205
Disinhibition	0.0921	0.0397	0.0207	0.0087
Perceived stress	−0.1678	0.0718	0.0198	8.56 E-03
Heart rate	−0.2502	0.1264	0.0481	0.0059
Fullness	1.0036	0.2458	<0.0001	0.0255

* *Variables differing at baseline between unsuccessful and successful responders*.

*See Table 1 for sample numbers*.

Changes induced by the LED intervention are shown in Table 4. As expected, there was a considerable difference in changes of fat mass, fat-free mass, percent body fat and waist circumference between groups of responders. The ANOVA also revealed a significant sex and sex by group interaction effect for fat mass, percent body fat, and waist circumference reflecting that changes were greater for men than women and that the differences between U and S responders were more pronounced in men. Accordingly, the decrease in resting heart rate as well as systolic and diastolic blood pressure was greater in S than in U responders.

**Table 4:** Changes induced by the intervention on outcome variables in the two groups of responders to LED.

**Variable**	**Group**	**Women**	**Men**	**p—sex**	**p—group**	**p—sex × group**
Fat-free mass (kg)	U S	−0.71 ± 3.94^*^−2.57 ± 2.24	−0.70 ± 3.43 −3.39 ± 2.95	0.0781	<0.0001	0.08
Fat mass (kg)	U S	−3.87 ± 4.34−7.54 ± 2.73	−4.48 ± 3.50 −9.62 ± 3.72	<0.0001	<0.0001	0.0146
Percent fat	U S	−1.95 ± 4.29−3.57 ± 2.68	−2.42 ± 3.58 −5.19 ± 3.28	0.0003	<0.0001	0.0484
Waist circumference (cm)	U S	−4.81 ± 5.26−9.22 ± 5.22	−4.87 ± 3.98 −11.7 ± 4.75	0.0046	<0.0001	0.0067
Systolic blood pressure (mmHg)	U S	−1.57 ± 11.8−6.90 ± 13.3	−3.50 ± 12.6 −9.63 ± 13.7	0.0616	<0.0001	0.8057
Diastolic blood pressure (mmHg)	U S	0.19 ± 8.77−2.62 ± 8.84	−1.07 ± 8.31 −5.86 ± 8.27	0.0045	<0.0001	0.2133
Heart rate (bpm)	U S	−2.08 ± 9.40−4.90 ± 8.88	−2.66 ± 7.65 −6.32 ± 9.55	0.2292	0.0001	0.6403
TFEQ restraint	U S	3.34 ± 3.613.05 ± 3.65	3.20 ± 4.63 3.70 ± 4.19	0.4908	0.7367	0.3322
TFEQ disinhibition	U S	−0.85 ± 2.82−1.06 ± 2.75	−1.10 ± 2.94 −1.21 ± 2.54	0.4125	0.4945	0.8874
TFEQ hunger	U S	−0.80 ± 3.17−1.02 ± 2.83	−0.25 ± 2.76 −1.25 ± 2.84	0.6294	0.0533	0.2081
PSS	U S	−0.88 ± 4.91−1.03 ± 5.40	−0.78 ± 5.99 −0.95 ± 5.09	0.8898	0.7615	0.9681
PSQI (n hours)	U S	−0.02 ± 0.83−0.01 ± 1.12	−0.36 ± 0.84 0.09 ± 1.05	0.2968	0.0385	0.0443
PSQI (total score)	U S	−0.91 ± 3.19−1.12 ± 2.78	−0.19 ± 2.13 −1.09 ± 2.63	0.2294	0.0508	0.2136
VAS-hunger (mm)	U S	−2.62 ± 31.5−10.4 ± 31.9	0.03 ± 27.2 −5.66 ± 31.8	0.2807	0.0501	0.759
VAS-satiety (mm)	U S	−14.9 ± 30.2−2.80 ± 29.8	−16.7 ± 30.7 −6.80 ± 27.7	0.3732	0.0006	0.7308
VAS-fullness (mm)	U S	−13.7 ± 33.3−10.4 ± 31.5	−7.57 ± 25.2 −16.9 ± 29.2	0.9342	0.3515	0.0539
VAS-thirst (mm)	U S	−2.52 ± 36.8−3.48 ± 31.0	−7.16 ± 23.4 −8.08 ± 28.2	0.1508	0.7497	0.9837
VAS-sweet (mm)	U S	−5.64 ± 29.6−21.9 ± 33.1	0.52 ± 31.7 −12.3 ± 28.9	0.021	<0.0001	0.6206
VAS-savory (mm)	U S	−2.35 ± 27.3−4.82 ± 29.5	2.10 ± 18.4 −1.77 ± 27.2	0.1964	0.3266	0.8582
VAS-desire to eat (mm)	U S	−2.02 ± 26.7−9.85 ± 30.4	−1.75 ± 25.3 −3.58 ± 25.1	0.2566	0.1251	0.298

* *Values are means ± SD. Analyses were performed on scores adjusted for age*.

*Group of responders: U and S refer to unsuccessful and successful responders, respectively*.

*See Table 1 for sample numbers*.

Assuming that the energy equivalent of fat and fat-free mass is 9,300 and 1,020 kcal/kg, respectively, and that the duration of the intervention was 56 days, it was estimated that the daily energy deficit was 656 and 1,299 kcal in female U and S responders, respectively (*p* < 0.01). The corresponding values for men were 815 and 1,659 kcal, respectively (*p* < 0.01). This inability of U responders to achieve an adequate daily energy deficit was accompanied by concordant changes related to appetite control. Specifically, there was a significant group effect reflecting more beneficial changes in S responders for susceptibility to hunger (TFEQ), sleep quality as well as hunger, satiety, and sweet sensations measured with VAS (Table 4).

The compliance score calculated from the sum of visits during the LED intervention was identical in U and S women (*n* = 4.7). In men, compliance was slightly lower in U than in S participants (*n* = 4.2 vs. 4.6).

## Discussion

The clinical experience in disease management frequently gives more attention to great achievements than to the profile of users who do not display the expected outcome. This is particularly obvious in the study of obesity where failure to succeed may be easily attributed to a lack of compliance, and even to gluttony and laziness. As described above, the relevant scientific literature contains evidence that the inability to reach a given clinical target may be explained by factors which are independent of the willingness of people to be compliant with specific guidelines (2, 3). In this regard, PREVIEW offered a unique opportunity to investigate this issue since the full participation in the study required the achievement of a minimal body weight loss during a short initial phase of LED intervention. As reported here, 10.2% of enrolled women and 7.9% of men having completed this study phase failed to lose 8% of their baseline body weight and were then excluded from the weight-maintenance phase. The characterization of their profile was the main preoccupation underlying the present study, the results of which allowed us to emphasize three aspects of their apparent resistance toward weight loss.

The first issue that was examined in unsuccessful weight responders was related to a state of baseline vulnerability. The results showed that they initially tended to be more overweight than successful responders. They also displayed higher levels of perceived stress, resting heart rate, and dietary restraint. They also reported lower levels of dietary disinhibition and perceived fullness. These observations agree with the results of Vogels and Westerterp-Plantenga (17) who used the same experimental approach to study the profile of unsuccessful and successful participants regarding their ability to prevent body weight regain after diet restriction. As in the present study, unsuccessful participants displayed increased restraint and lower disinhibition at baseline compared to successful controls. To ascertain the clinical relevance of these observations, correlation analyses were performed to determine the extent to which these variations predicted the main outcome of the LED intervention, i.e., percent body weight loss. Interestingly, significant negative associations were observed between the baseline levels of perceived stress, resting heart rate or dietary restraint and body weight changes. These represented a cluster of relationships between stress-related variables and body weight loss. Indeed, the perceived stress scale provided an indication of individual stress, resting heart rate is a physiological variable that is sensitive to stress whereas dietary restraint may either reflect the consequence or a source of stress promoting inadequate changes in food intake. This is concordant with studies having shown an association between dietary restraint and cortisol, be it measured in saliva (18, 19) or urine (20). From a mechanistic standpoint, these observations remind that some decades ago, adrenalectomy was considered as a preventive approach of obesity and the increased sensitivity of obese experimental animals to glucocorticoids was documented (21, 22). This agrees with subsequent genetic studies in humans having shown that the polymorphism of the glucocorticoid receptor gene (GRL IVS2-BclI) is related to visceral fat deposition (23) and long-term body weight/fat gain (24). Globally, the results of the present study are concordant with the idea that the risk of overweight may be linked to stress vulnerability. They also support the concept that the weight loss response to LED at least partly depends on factors which are independent of the spontaneous motivation of participants.

It is well-established that body weight/fat loss promotes biological/behavioral adaptations that are susceptible to induce weight regain. These changes include a greater than predicted decrease in energy expenditure (25–27), an increase in hunger, desire to eat and energy intake (28, 29), and concordant changes in relevant biomarkers such as plasma leptin (28, 30) and ghrelin (31, 32). Accordingly, Tremblay et al. (3) reported that female low body weight responders to diet restriction exhibited a low reduction of susceptibility to hunger in comparison to high weight losers. In this regard, the present study offered the opportunity to document a second aspect of resistance to weight loss being related to the possibility that LED intervention may induce less favorable behavioral effects in unsuccessful than in successful weight responders. As indicated above, both female and male unsuccessful responders were clearly unable to maintain an adequate daily energy deficit during the LED intervention. This limitation was accompanied by changes in appetite control which were susceptible to compromise subsequent body weight stability. Specifically, unsuccessful weight responders tended to report a lesser improvement in susceptibility to hunger measured with the TFEQ. Furthermore, even if the measurements of appetite sensations with VAS at a single point in a free-living context may represent a limitation, they nevertheless provided a concordant discrimination between U and S responders. Indeed, the use of VAS revealed less beneficial or detrimental changes in hunger, satiety and sweet taste compared to successful responders. Thus, these observations globally suggest that LED may accentuate the behavioral vulnerability of unsuccessful body weight responders.

The results of this study also permitted to determine if unsuccessful weight responders had a reduced ability to benefit from a LED intervention regarding some factors related to body weight stability. To this effect, it is relevant to consider changes in sleep quality and duration that were found to be related to the body fat loss induced by a diet-based weight reducing program (16). The present study showed that there was a significant between-group difference for changes in both sleep quality and duration which were more beneficial in successful responders. Furthermore, the decrease in sleep duration observed in unsuccessful responders was more pronounced in men, as reflected by the significant sex X group interaction effect. This is concordant with the fact that the between-group difference in fat loss was more pronounced in men and the observation that a decrease in sleep duration predicts a reduced outcome in fat loss to a diet-based weight-reducing program (16). This is also in agreement with the well-established sex difference in weight loss that was confirmed in the PREVIEW intervention (6) and that was observed in our previous research (33).

In summary, the results of this study showed that 8 to 10% of subjects enrolled in PREVIEW displayed a response in body weight that was insufficient to pursue the protocol after the initial phase of LED intervention. They were characterized by a state of baseline vulnerability for some behavioral variables which were related to percent weight loss. Moreover, it seems that their vulnerability related to appetite control was accentuated by diet restriction even if the energy deficit they could achieve was much smaller than that of successful responders. Finally, unsuccessful weight responders were less able to benefit from the intervention regarding some favorable effects of the intervention such as an improvement of sleep quality. Globally, these results support the hypothesis of potential unfavorable effects of LED in some individuals and they also highlight the need for personalized nutrition interventions allowing to increase the compatibility between diet prescription and what the body and mind are able to tolerate.

## Data Availability Statement

The raw data supporting the conclusions of this article will be made available by the authors, without undue reservation.

## Ethics Statement

The studies involving human participants were reviewed and approved by (clinicaltrials.gov NCT01777893). The patients/participants provided their written informed consent to participate in this study.

## Author Contributions

The PREVIEW project was designed by AR, JB-M, MW-P, MF, WS, and EF. The protocol for the PREVIEW adult intervention study was written by MF, TLar, and AR. MW-P, IM, JM, SP, WS, GS, and SH were involved in developing the study design. AT analyzed and interpreted data and drafted the manuscript. All authors contributed to the implementation of the experimental trial, contributed to analysis and interpretation of the data, contributed to critical revision of the manuscript for important intellectual content, agreed that the accuracy and integrity of the work has been appropriately investigated and resolved, and approved the final version of the manuscript. AT had full access to the data, had final responsibility for the decision to submit for publication, attests that all listed authors meet authorship criteria, and that no others meeting the criteria have been omitted.

## Funding

This study received grants from the EU 7th Framework Programme (FP7-KBBE-2012), grant agreement No. 312057, the New Zealand Health Research Council, grant No. 14/191, and the NHMRC-EU Collaborative Grant, Australia. All LED products were provided by Cambridge Weight Plan, UK. AT is partly funded by the Canada Research Chair in Environment and Energy Balance.

## Conflict of Interest

TL is employed by NetUnion sarl, who contributed to the data collection process in the absence of commercial or financial conflict of interest with the study analysis. The remaining authors declare that the research was conducted in the absence of any commercial or financial relationships that could be construed as a potential conflict of interest.

## Publisher's Note

All claims expressed in this article are solely those of the authors and do not necessarily represent those of their affiliated organizations, or those of the publisher, the editors and the reviewers. Any product that may be evaluated in this article, or claim that may be made by its manufacturer, is not guaranteed or endorsed by the publisher.
